# Effect of Selenium Treatment on Central Insulin Sensitivity: A Proteomic Analysis in β-Amyloid Precursor Protein/Presenilin-1 Transgenic Mice

**DOI:** 10.3389/fnmol.2022.931788

**Published:** 2022-07-07

**Authors:** Xia Xu, Pishui Qi, Ying Zhang, Huihuan Sun, Yong Yan, Wenxiu Sun, Shudong Liu

**Affiliations:** ^1^Department of Nursing, School of Nursing, Shandong Xiehe University, Jinan, China; ^2^Department of Pharmacy, Shandong Rongjun General Hospital, Jinan, China; ^3^Department of Endocrinology, Shandong Rongjun General Hospital, Jinan, China; ^4^Department of Transfusion Medicine, Shandong Provincial Hospital Affiliated to Shandong First Medical University, Jinan, China; ^5^Department of Pharmacy, Taishan Vocational College of Nursing, Taian, China

**Keywords:** Alzheimer’s disease (AD), selenium, insulin sensitivity, proteomic analysis, neurodegenerative disease, metabolic diseases

## Abstract

Prior studies have demonstrated a close association between brain insulin resistance and Alzheimer’s disease (AD), while selenium supplementation was shown to improve insulin homeostasis in AD patients and to exert neuroprotective effects in a mouse model of AD. However, the mechanisms underlying the neuroprotective actions of selenium remain incompletely understood. In this study, we performed a label-free liquid chromatography-tandem mass spectrometry (LC–MS/MS) quantitative proteomics approach to analyze differentially expressed proteins (DEPs) in the hippocampus and cerebral cortex of Aβ precursor protein (APP)/presenilin-1 (PS1) mice following 2 months of treatment with sodium selenate. A total of 319 DEPs (205 upregulated and 114 downregulated proteins) were detected after selenium treatment. Functional enrichment analysis revealed that the DEPs were mainly enriched in processes affecting axon development, neuron differentiation, tau protein binding, and insulin/insulin-like growth factor type 1 (IGF1)-related pathways. These results demonstrate that a number of insulin/IGF1 signaling pathway-associated proteins are differentially expressed in ways that are consistent with reduced central insulin resistance, suggesting that selenium has therapeutic value in the treatment of neurodegenerative and metabolic diseases such as AD and non-alcoholic fatty liver disease (NAFLD).

## Introduction

Growing evidence implicates abnormal insulin signaling in the pathogenesis of Alzheimer’s disease (AD), a neurodegenerative condition whose fundamental feature is β-amyloid (Aβ) and tau protein deposition in the brain (De La Monte and Wands, 2008; Ahmad, 2012; Jahangir et al., 2014; Sridhar, 2015; Ribes-Navarro et al., 2018). AD represents a global public health problem; a recent study indicated that its prevalence in Chinese adults aged 60 years and older is ∼3.9% (Jia et al., 2020). AD is characterized by progressive cognitive decline, memory loss, sleep disturbance, and neuropsychiatric symptoms. It is the leading cause of dementia worldwide and is associated with significant reductions in lifespan and quality of life (Greer et al., 2013). Both genetic and environmental factors contribute to AD neuropathology; this is exacerbated by the interaction between Aβ precursor protein (APP) and fibrillar Aβ, which enhances cytotoxicity and triggers neuroinflammation by activating microglia and astrocytes (Lorenzo et al., 2000; Ozben and Ozben, 2019).

A number of clinical and experimental studies indicated that both central and peripheral insulin resistance influence the development of AD (Neth and Craft, 2017; Chow et al., 2019). It has been shown that the brain response to peripheral insulin declines in older people, and that low cerebrospinal fluid and high plasma insulin levels are characteristically found in AD patients (Craft et al., 1998; Deli et al., 2015). In this regard, impairment of insulin transport into the central nervous system, as well as abnormal expression and dysfunction of insulin receptor substrate 1 (Irs1), insulin receptor (InsR), insulin-like growth factor type 1 (IGF1), IGF1 receptor (IGFR), and insulin-degrading enzyme (IDE), were proposed to contribute to brain insulin resistance and AD development (Steen et al., 2005; Jahangir et al., 2014; Ribes-Navarro et al., 2018; Taguchi et al., 2019; Kellar and Craft, 2020). Based on the above evidence, and although results were modest, clinical trials evaluating the efficacy of insulin sensitizers such as metformin and rosiglitazone suggested improvement in insulin sensitivity and cognitive impairment in AD patients (Kellar and Craft, 2020). Along these lines, liraglutide (a glucagon-like peptide-1 receptor agonist) was shown to increase peripheral insulin sensitivity, attenuate memory impairment, and reduce amyloid plaque deposition in aged transgenic APP/presenilin-1 (PS1) mice, a widely used animal model of AD (Mcclean and Hölscher, 2014).

Selenium is an essential micronutrient in mammals and exerts multiple biological (e.g., antioxidant, anti-inflammatory, and immunomodulatory) activities (Mangiapane et al., 2014; Rayman, 2019). The impact of selenate on cellular physiology is concentration dependent, and its deficiency or excess is involved in pathophysiological processes related to multiple conditions and diseases, including aging, infertility, viral infection, thyroid disorders, and AD (Loef et al., 2011; Mintziori et al., 2019; Gheorghiu and Badiu, 2020; Kieliszek, 2021). Thus, the therapeutic potential of selenium supplementation is of great clinical interest (Wang et al., 2017). Clinical evidence indicates that selenium levels are decreased in the hippocampal, temporal, and cortical regions of AD patients, which is consistent with reduced antioxidant capacity and increased oxidative stress (Varikasuvu et al., 2018). A few clinical trials evaluating the effect of selenium supplementation on AD symptoms showed promising results, evidenced by lowered insulin and triglyceride levels, increased antioxidant capacity, and improvements in some markers of cognitive function (Tamtaji et al., 2019; Ashraf and So, 2020). Consistent with clinical findings, a recent study demonstrated that sodium selenate treatment improved cognitive impairment and neuropsychiatric symptoms by reducing the accumulation of tau protein in the hippocampus of PS1/APP/tau triple transgenic AD model (3xTg-AD) mice (Van Der Jeugd et al., 2018).

Despite the evidence summarized above, the specific impact of selenium on the expression of insulin signaling-related proteins in the brains of AD patients and animal models remains unknown. To address this issue, we performed label-free liquid chromatography-tandem mass spectrometry (LC–MS/MS) quantitative proteomics analysis of the hippocampus and cerebral cortex of transgenic APP/PS1 mice treated with sodium selenate. Our findings may provide new directions for the improved treatment or prevention of AD and other neurodegenerative disorders.

## Materials and Methods

### Mice

Transgenic APP/PS1 mice were purchased from Nanjing Junke Bioengineering Co., Ltd. (Nanjing, China). Mice were housed in standard laboratory conditions with constant humidity (55%) and temperature (22 ± 2°C) under a 12-h light-dark cycle with *ad libitum* access to water and food. Twelve-month-old male APP/PS1 transgenic mice were randomly divided into treatment and control groups (*n* = 10 in each group) and treated with 12 μg/ml sodium selenate (Sigma–Aldrich/Merck, St. Louis, MO, United States) or without sodium selenate in drinking water for 2 months (Van Der Jeugd et al., 2018). The experimental procedures were performed and approved by the Animal Care and Use Committee of Shandong Xiehe University (Jinan, China). After treatment, the mice were euthanized, and the hippocampus and cerebral cortex were immediately excised for subsequent proteomic analysis.

### Proteomics

#### Sample Preparation and Liquid Chromatography Tandem Mass Spectrometry

The brain tissue was homogenized with SDT buffer (4% SDS, 100 mM Tris–HCl, 1 mM DTT, pH 7.6) for sample lysis and protein extraction (Zhu et al., 2014). Protein was quantified with a bicinchoninic acid (BCA) Protein Assay Kit (Bio-Rad, Laboratories, Hercules, CA, United States).

Protein samples were digested into peptides using the filter-aided sample preparation (FASP) method as previously described (Wiśniewski, 2018). LC–MS/MS analysis was performed on a timsTOFPro mass spectrometer coupled to a nanoElute UHPLC system (Bruker Daltonics, Germany) with a run time of 60 min. The peptides were loaded onto a reversed-phase trap column (Thermo Scientific Acclaim PepMap100, 100 μm × 2 cm, nanoViper C18) connected to a C18 reversed-phase analytical column (Thermo Scientific Easy Column, 10 cm long, 75 μm inner diameter, 3 μm resin) in buffer A (0.1% formic acid) and separated with a linear gradient of buffer B (84% acetonitrile and 0.1% formic acid) at a flow rate of 300 nl/min controlled by IntelliFlow technology. The mass spectrometer was operated in positive ion mode, collected ion mobility MS spectra over a mass range of m/z 100–1,700 and 1/k0 of 0.6 to 1.6, and then performed 10 cycles of PASEF MS/MS with a target intensity of 1.5k and a threshold of 2,500. Active exclusion was enabled with a release time of 0.4 min.

#### Protein Identification and Quantification

For identification and quantification analysis, raw MS sample data were combined and analyzed using MaxQuant 1.5.3.17 software (Chen et al., 2019). MS data were matched against the UniProt_MusMusculus_17027_20200226 database. The label-free quantification algorithm was used for quantitative analysis. Proteins with an adjusted *P*-value < 0.05 and fold change >1.5 were considered as significant.

#### Gene Ontology Annotation

The sequences of the selected DEPs were locally aligned using NCBI BLAST+ client software (ncbi-blast-2.2.28+-win32.exe) and InterProScan to find homologous sequences. Then, Gene Ontology (GO) terms were mapped, and sequences were annotated using Blast2GO software. GO annotation results were plotted with R scripts.

#### Kyoto Encyclopedia of Genes and Genomes Annotation

Following annotation steps, the studied proteins were blasted against the online Kyoto Encyclopedia of Genes and Genomes (KEGG) database^1^ to retrieve their orthology identifications and corresponding pathways. GO and KEGG pathway enrichment analyses were applied based on Fisher’s exact test, considering all quantified proteins as the background dataset.

#### Protein–Protein Interaction Network

The protein–protein interaction (PPI) network of the identified differentially expressed proteins (DEPs) was retrieved from the IntAct database.^2^ Cytoscape software (version 3.6.1) was utilized to visualize and analyze potential correlations between DEPs (Shannon et al., 2003).

#### RNA Isolation and Real-Time Quantitative Reverse Transcribed Polymerase Chain Reaction

Total RNA was isolated from the hippocampus and cortex of mice using a TRIzol kit (Invitrogen, Carlsbad, CA, United States) and reverse-transcribed to cDNA using PrimeScript reagent (TaKaRa, Kusatsu, Japan) following the manufacturer’s instructions. SYBR Green Premix Ex TaqII (TaKaRa, Kusatsu, Japan) and the Roche 480 detection system were used to analyze the relative mRNA expression of target genes. GAPDH was used as an internal control for normalization. The relative gene expression levels were calculated according to the 2^–ΔΔCt^ method.

The primer sequences were as follows:

mouse Irs1 forward primer 5′-TCTACACCCGAGACGAAC ACT-3′, Irs1 reverse primer 5′-TGGGCCTTTGCCCGATTATG-3′; InsR forward primer 5′-TCAAGACCAGACCCGAAGATT-3′, InsR reverse primer 5′-TCTCGAAGATAACCAGGGCATAG-3′; serine/arginine-rich splicing factor (Srsf3) forward primer 5′-CTGTGTGGCTGCCGTGTAA-3′, Srsf3 reverse primer, 5′-TCCTCCTGCGGTAATCATCTC-3′; growth factor receptor-bound protein 2 (Grb2) forward primer 5′-ACGGAGCCGGGAAGTATTTC-3′, Grb2 reverse primer, 5′-GGTTCCTGGACACGGATGTTG-3′; GAPDH forward primer 5′-GTCGGTGTGAACGGATTTG-3′, and GAPDH reverse primer 5′-GAATTTGCCGTGAGTGGAG-3′.

### Immunohistochemistry

The immunohistochemistry protocol was conducted as described previously (Eraky et al., 2021). Briefly, paraffin sections (5 μM thick) of the hypothalamus were deparaffinized, rehydrated, and then immunostained with rabbit Irs-1 (Servicebio, Wuhan, China) polyclonal and Srsf3 monoclonal antibodies (Thermo Fisher Scientific, Runcorn, Cheshire, United Kingdom). After three washes in phosphate-buffered saline (pH 7.4), slides were subsequently incubated with corresponding species of horseradish peroxidase-labeled secondary antibody. Diaminobenzidine (Servicebio, Wuhan, China) was incubated as a chromogenic reagent. The staining time was controlled under the microscope. Positive staining was brownish yellow. Hematoxylin was used for counterstaining and visualized by a Nikon optical microscope (Nikon, Tokyo, Japan). Average optical density (AOD) value was used to semi-quantify the protein expression on immunohistochemical staining by ImageJ software (version 1.45) (NIH, Bethesda, MD, United States) (Liu et al., 2015).

### Statistical Analysis

All data were expressed as the mean ± SEM and analyzed by Prism v 6.0 (GraphPad Software Inc., San Diego, CA, United States). A two-tailed, unpaired Student’s *t*-test was used to examine the significant difference between two groups. *P* < 0.05 was considered as statistically significant. All experiments were conducted three times.

## Results

### Selenium Supplementation Induces Differential Protein Expression in the Aβ Precursor Protein/Presenilin-1 Mouse Brain

**FIGURE 1:**
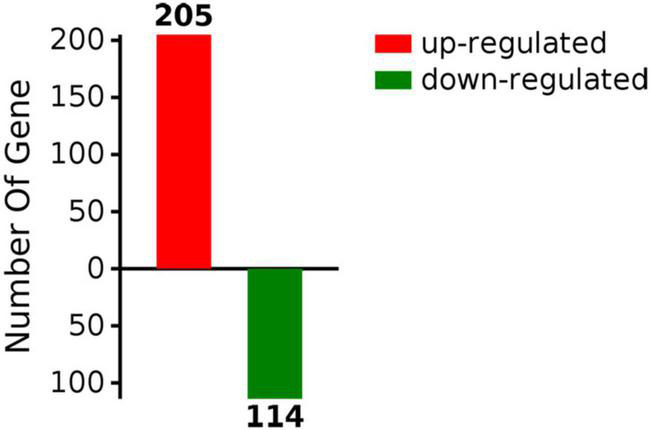
Differentially expressed proteins in brain tissue of sodium selenate-treated APP/PS1 mice.

There is a close association between brain insulin resistance and AD (Sridhar, 2015). Because selenium supplementation was shown to improve insulin homeostasis in AD patients and to exert neuroprotective effects in a mouse model of AD (Sridhar, 2015; Van Der Jeugd et al., 2018; Tamtaji et al., 2019), we performed label-free LC–MS/MS quantitative proteomics to identify DEPs in the hippocampus and cerebral cortex of APP/PS1 mice—an AD animal model—following 2 months of treatment with sodium selenate. A total of 2,389 proteins were identified by proteomics analysis; among these, 319 DEPs (>1.5-fold change), including 205 upregulated and 114 downregulated proteins, were detected in the brains of selenate-treated mice compared to control mice (Figure 1). These data indicate that sodium selenate treatment has a distinct impact on brain protein expression in AD mice.

### Gene Ontology Analysis of Differentially Expressed Proteins

**FIGURE 2:**
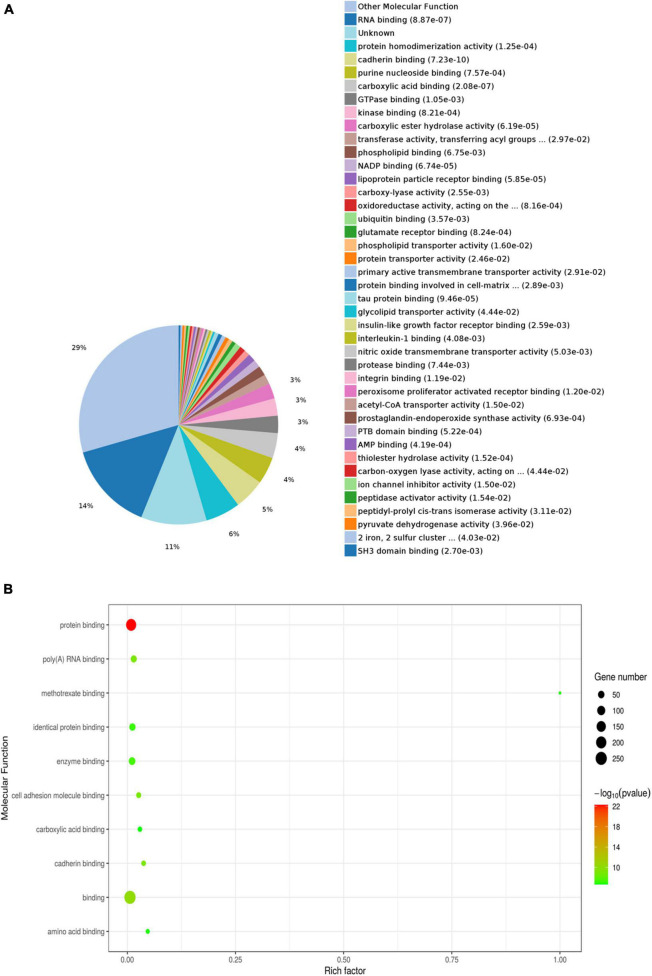
Gene Ontology (GO) enrichment analysis of molecular function for selenium-influenced DEPs. **(A)** Pie chart of DEP-enriched GO terms for molecular function (MF). **(B)** Enriched GO terms for MF.

**FIGURE 3:**
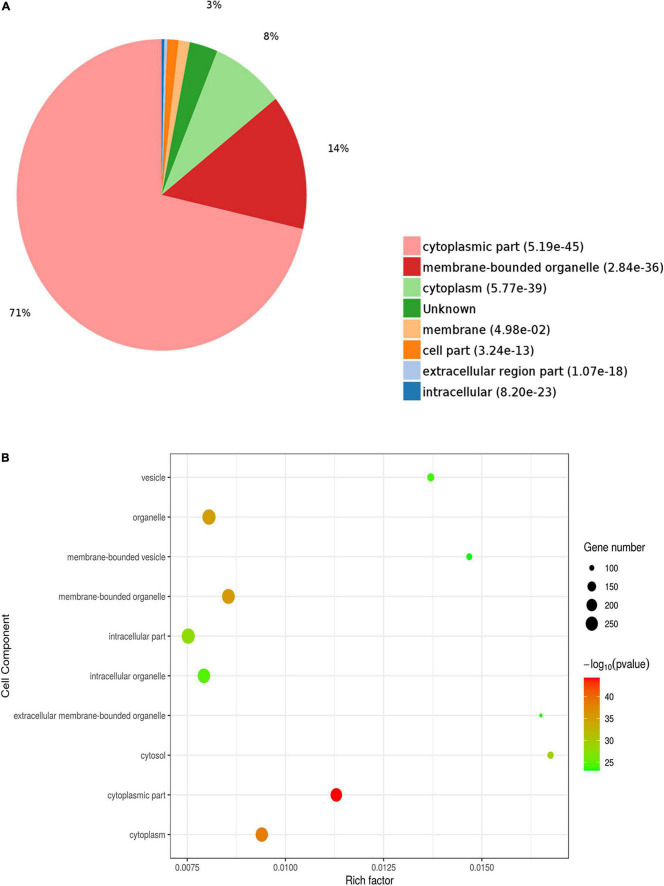
Gene Ontology (GO) enrichment analysis of cellular components for selenium-influenced DEPs. **(A)** Pie chart of DEP-enriched GO terms for cellular component (CC). **(B)** Enriched GO terms for CC.

To investigate the functions of the DEPs influenced by sodium selenate treatment, GO and KEGG enrichment analyses were conducted. In the molecular function (MF) category, the DEPs were mainly enriched in “RNA binding,” “protein homodimerization activity,” “cadherin binding,” “insulin-like growth factor receptor binding,” and “tau protein binding” (Figures 2A,B). The latter term suggests a differential impact of selenate in AD pathophysiology by influencing tau dynamics (Jouanne et al., 2017). Within the cellular component (CC) category, the DEPs were mainly enriched in “cytoplasmic part” (71%), “membrane-bounded organelle” (14%), and “cytoplasm” (8%) (Figures 3A,B).

**FIGURE 4:**
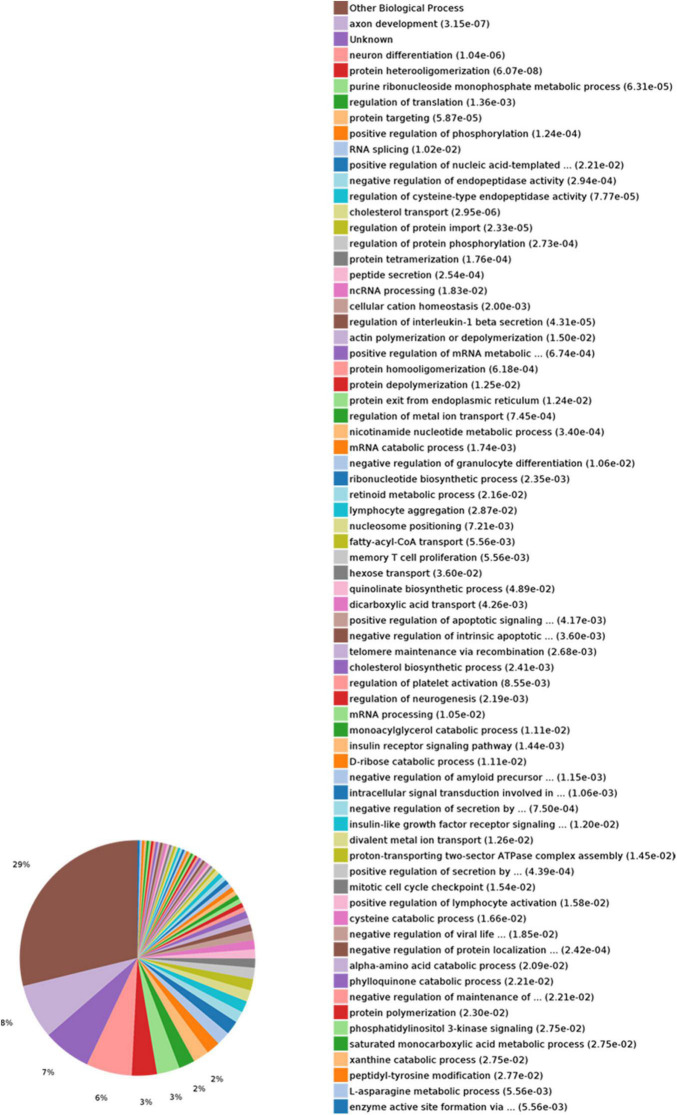
Gene Ontology (GO) enrichment analysis of biological processes for selenium-influenced DEPs.

**FIGURE 5:**
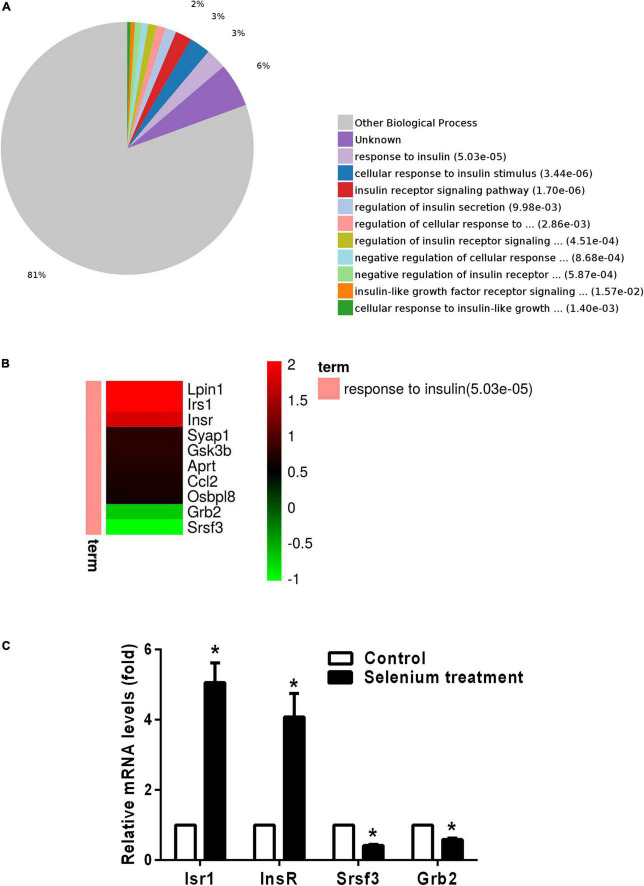
Gene Ontology (GO) enrichment analysis of biological processes for DEPs involved in insulin signaling pathways. **(A)** Pie chart of DEP-enriched GO terms for biological processes related to the insulin/IGF signaling pathway. **(B)** Heatmap of upregulated and downregulated DEPs involved in the response to insulin. **(C)** The relative mRNA levels of Irs1, InsR, Srsf3, and Grb2 in the cortex of APP/PS1 mice treated with or without selenium. GAPDH was used for normalization, and the control was set to 1 in the RT-PCR data. All experiments were performed in duplicate. *P < 0.05 compared with control (untreated).

**FIGURE 6:**
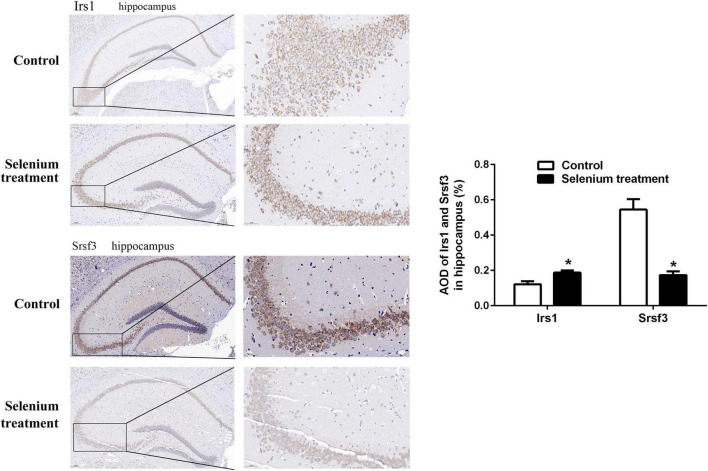
Immunohistochemistry staining (at higher magnification) of Irs1 and Srsf3 expression in the hippocampus of APP/PS1 mice treated with or without selenium. AOD value is calculated by ImageJ. *P < 0.05 compared with control (*n* = 4 per group). The positive staining is brownish yellow. Magnification 200×, scale bar = 100 μM.

**FIGURE 7:**
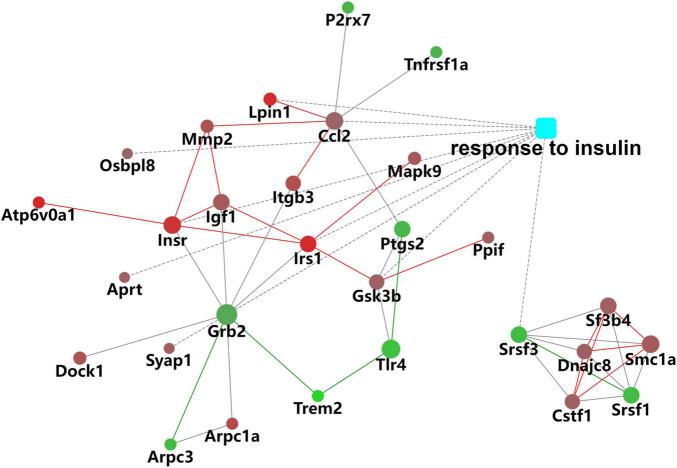
Protein–protein interaction (PPI) network of DEPs associated with the response to insulin.

In the biological process (BP) category, the identified DEPs showed enrichment in axon development (8%) and neuron differentiation (6%) (Figure 4). In addition, 3, 3, and 2% of DEPs were associated with “response to insulin,” “cellular response to insulin stimulus,” and “insulin receptor (IR) signaling pathway,” respectively, whereas other DEPs were implicated in “cellular response to insulin-like growth factor stimulus,” “insulin-like growth factor receptor signaling pathway,” and “regulation of insulin secretion” (Figure 5A). In relation to the response to insulin, eight DEPs, including the candidate phosphatase LPIN1 (Lpin1), Irs1, and InsR, were upregulated, while two DEPs (Srsf3 and Grb2) were downregulated after sodium selenate treatment (Figure 5B and Table 1). Validation of significantly DEPs by real-time quantitative reverse transcribed polymerase chain reaction (RT-PCR) assay were consistent with the results in the proteomic approach (Figure 5C). Similarly, there were a significant increased expression of Irs1 and a decreased expression of Srsf3 by immunohistochemistry staining in the hippocampus of APP/PS1 mice after selenium treatment compared with the untreated control mice (Figure 6). In turn, PPI network analysis revealed that Grb2 may interact with InsR, Irs1, and IGF1 (Figure 7). These results suggest that selenium supplementation exerts a major effect on the regulation of central insulin sensitivity.

**TABLE 1:** Identification of selenium-induced DEPs associated with the response to insulin.

Change	Protein ID	Protein name	Gene name	Fold change
Up	Q91ZP3	Phosphatidate phosphatase LPIN1	Lpin1	4.11
Up	P35569	Insulin receptor substrate 1	Irs1	4.09
Up	P15208	Insulin receptor	InsR	3.49
Up	Q9D5V6	Synapse-associated protein 1	Syap1	1.70
Up	Q9 WV60	Glycogen synthase kinase-3 beta	Gsk3b	1.69
Up	P08030	Adenine phosphoribosyltransferase	Aprt	1.63
Up	P10148	C-C motif chemokine 2	Ccl2	1.59
Up	B9EJ86	Oxysterol-binding protein-related protein 8	Osbpl8	1.53
Down	Q60631	Growth factor receptor-bound protein 2	Grb2	0.61
Down	P84104	Serine/arginine-rich splicing factor 3	Srsf3	0.49

*Up, upregulated; down, downregulated.*

### Kyoto Encyclopedia of Genes and Genomes Pathway Analysis of Differentially Expressed Proteins

**FIGURE 8:**
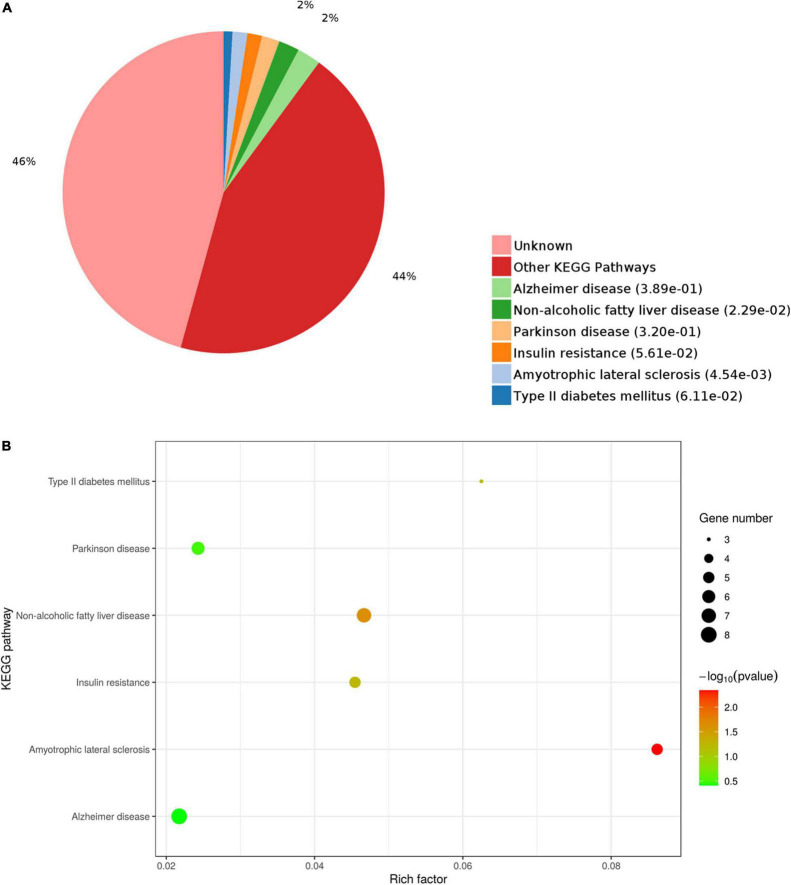
Kyoto Encyclopedia of Genes and Genomes (KEGG) pathway analysis of DEPs. **(A)** Pie chart of DEP-enriched KEGG pathways. **(B)** KEGG pathway enrichment distribution of DEPs.

The functions of the identified DEPs were further explored by KEGG pathway enrichment analysis. The results showed enrichment of DEPs in pathways related to several pathological conditions, including AD, non-alcoholic fatty liver disease (NAFLD), Parkinson’s disease, insulin resistance, amyotrophic lateral sclerosis, and type II diabetes mellitus (Figures 8A,B). These data further support the influence of selenium supplementation on the expression of proteins closely associated with neurodegenerative and metabolic diseases.

## Discussion

A growing body of research indicates that impaired insulin/IGF signaling contributes to brain insulin resistance and plays a causal role in AD pathogenesis (De La Monte and Wands, 2008; Ahmad, 2012; Sridhar, 2015; Neth and Craft, 2017; Denver et al., 2018; Song et al., 2018; Komleva et al., 2021). Insulin resistance promotes metabolic dysfunction, oxidative stress, and inflammation and is a main determinant of the proven association between diabetes and AD development (De La Monte and Wands, 2008; Chow et al., 2019; Spinelli et al., 2019, 2020). Regarding the relevance of the APP/PS1 transgenic mouse as an experimental AD model, a previous study showed that impaired glucose tolerance and reduced insulin sensitivity precede amyloid plaque deposition and cognitive decline in these mice (Macklin et al., 2017).

Growing research indicates that selenium supplementation may have beneficial effects on AD progression and symptoms (Tamtaji et al., 2019). Importantly, clinical trials showed that selenium supplementation regimens were well tolerated, improved insulin homeostasis, and slowed disease progression in AD patients (Malpas et al., 2016; Tamtaji et al., 2019; Vivash et al., 2021). However, the molecular mechanisms underlying the neuroprotective actions of selenium remain incompletely understood.

In this study, we performed a label-free LC–MS/MS quantitative proteomics approach to analyze selenium-induced changes in protein expression in the hippocampus and cerebral cortex of APP/PS1 mice. Suggestive of the extensive effects of selenium on AD pathophysiology, 319 DEPs (205 upregulated and 114 downregulated proteins) were detected after sodium selenate treatment. Importantly, functional enrichment analysis revealed that the DEPs were mainly enriched in processes affecting tau protein binding and insulin-related pathways, which are significantly related to the onset and progression of AD (Oddo et al., 2003; Jouanne et al., 2017).

Specifically, GO analysis showed the involvement of the identified DEPs in BPs such as “axon development,” “neuron differentiation,” “response to insulin,” “cellular response to insulin stimulus,” “insulin receptor signaling pathway,” “IGFR signaling pathway,” and “regulation of insulin secretion.” Several DEPs related to the insulin response, including Lpin1, Irs1, and InsR, were significantly upregulated in the brains of AD mice following sodium selenate treatment.

Phosphatidate phosphatase LPIN1 plays an important role in adipocyte differentiation and lipid metabolism; decreased Lpin1 expression is correlated with insulin resistance, and mutations in Lpin1 determine cognitive impairment in mice with fatty liver dystrophy (Ryu et al., 2009; Chen et al., 2012; Shang et al., 2020; Xie et al., 2020). Lpin1 expression is decreased in the hippocampus of a rat model of type I diabetic encephalopathy. In this model, lentivirus-mediated lipin1 overexpression was able to improve cognitive deficits and exert neuroprotective effects by inhibiting the protein kinase D/limk1/cofilin signaling pathway (Xie et al., 2020). However, because the association between Lpin1 and AD remains uncertain, further work is needed to assess whether Lpin1 upregulation contributes to the beneficial effect of selenium against AD.

Decreased expression of both InsR and IGFR was observed in the cortex and hippocampus of AD patients (Steen et al., 2005). A recent study demonstrated that sodium selenate supplementation exerted a beneficial effect on healthspan and metabolism by inhibiting IGF1 signaling and other hormones (Plummer et al., 2021). Moreover, in an AD mouse model, IGF1 overexpression led to decreased cortical and hippocampal expression of amyloid β protein (Aβ1–40) (Song et al., 2018). Hence, both human and animal research strongly suggest that strategies aimed at restoring insulin sensitivity may be effective in decreasing amyloid deposition (Han et al., 2017; Khandelwal et al., 2020). In this regard, our findings suggest that the upregulation of Irs1 and InsR and the improvement of insulin/IGF signaling are likely important determinants of the beneficial effects of selenium-based therapies targeting AD.

Our study also showed that the levels of Srsf3 and Grb2, two proteins also linked to insulin responses, were significantly downregulated after treatment with sodium selenate. Srsf3 is critical for mRNA splicing and mRNA nuclear export (Xiao et al., 2016; Nakura et al., 2021). A recent study demonstrated that Srsf3 was involved in alternative splicing of the InsR gene to produce two variants, InsR-A and InsR-B, which display different tissue specificity and regulatory roles (Nakura et al., 2021). Another study revealed that Srsf3 expression is increased in the hippocampus and temporal cortex of AD patients and suggested that its activity modulates tropomyosin receptor kinase B (TrkB) pre-mRNA splicing to promote neurodegeneration (Wong et al., 2012). Furthermore, Srsf3 was also shown to affect alternative splicing of the tau gene, one of the main biomarkers of AD (Yu et al., 2004; Peyron et al., 2021). The above observations are in line with our study in suggesting that inhibition of Srsf3 expression mediates, at least in part, the beneficial effect of selenium on AD pathology.

Growth factor receptor-bound protein 2 is a key adaptor protein in the insulin signaling pathway (Kasuga, 1993; Liu et al., 2009). Previous studies reported that Grb2 expression is upregulated in the brains of AD patients and model mice and suggested that Grb2 contributes to brain insulin resistance and AD pathogenesis through interactions with APP and NADPH oxidase 4 (Nox4) (Bruce-Keller et al., 2011; Wu and Williams, 2012; Roy et al., 2014; Majumder et al., 2017, 2021; Park et al., 2021). Notably, downregulation of Grb2 in skeletal muscle was suggested to mediate the insulin-sensitizing effect of caloric restriction, a notion supported by data from both Grb2^±^ heterozygous knockout mice and muscle cells *in vitro* (Liu et al., 2009). Therefore, downregulation of Grb2 may represent another mechanism by which selenium helps improve insulin signaling in AD.

There were several limitations in our study. However, one of the most important limitations is that the study protocol lacked the inclusion of healthy controls and a group treated with selenium alone. In the absence of these groups, the generalizability of these results is challenging and warrants further investigation.

Taken together, our present study demonstrates that several insulin/IGF1 signaling pathway-associated proteins are differentially expressed in ways that are consistent with reduced central insulin resistance after treatment of APP/PS1 mice with sodium selenate. Although risk-assessment principles should guide the clinical administration of selenium-based therapies, our findings support their therapeutic value in the treatment of neurodegenerative and metabolic diseases such as AD and NAFLD.

## Data Availability Statement

The datasets presented in this study can be found in online repositories. The name of the repository and accession number can be found below: iProX, https://www.iprox.cn/, IPX0004556000. The mass spectrometry proteomics data have been deposited to the ProteomeXchange Consortium (http://proteomecentral.proteomexchange.org) *via* the iProX partner repository (Ma et al., 2019) with the dataset identifier PXD034613.

## Ethics Statement

The animal study was reviewed and approved by the Animal Care and Use Committee of Shandong Xiehe University (Jinan, China).

## Author Contributions

WS and SL conceived the idea and designed the study. XX, PQ, and YY carried out the animal experiments. YZ and HS performed the proteomics work. XX conducted the formal analysis and prepared the manuscript. SL was responsible for the review and revision of the manuscript. All authors have read and agreed to the published version of the manuscript.

## Conflict of Interest

The authors declare that the research was conducted in the absence of any commercial or financial relationships that could be construed as a potential conflict of interest.

## Publisher’s Note

All claims expressed in this article are solely those of the authors and do not necessarily represent those of their affiliated organizations, or those of the publisher, the editors and the reviewers. Any product that may be evaluated in this article, or claim that may be made by its manufacturer, is not guaranteed or endorsed by the publisher.

## Footnotes

1
http://geneontology.org/

2
http://www.ebi.ac.uk/intact/main.xhtml
